# Genomewide Association Scan of Suicidal Thoughts and Behaviour in Major Depression

**DOI:** 10.1371/journal.pone.0020690

**Published:** 2011-07-05

**Authors:** Alexandra Schosser, Amy W. Butler, Marcus Ising, Nader Perroud, Rudolf Uher, Mandy Y. Ng, Sarah Cohen-Woods, Nick Craddock, Michael J. Owen, Ania Korszun, Lisa Jones, Ian Jones, Michael Gill, John P. Rice, Wolfgang Maier, Ole Mors, Marcella Rietschel, Susanne Lucae, Elisabeth B. Binder, Martin Preisig, Julia Perry, Federica Tozzi, Pierandrea Muglia, Katherine J. Aitchison, Gerome Breen, Ian W. Craig, Anne E. Farmer, Bertram Müller-Myhsok, Peter McGuffin, Cathryn M. Lewis

**Affiliations:** 1 MRC Social Genetic and Developmental Psychiatry Centre, Institute of Psychiatry, King's College London, London, United Kingdom; 2 Department of Psychiatry and Psychotherapy, Medical University Vienna, Vienna, Austria; 3 Department of Psychiatry, University of Hong Kong, Hong Kong, Special Administrative Region, China; 4 Max Planck Institute of Psychiatry, Munich, Germany; 5 Department of Psychiatry, University of Geneva, Geneva, Switzerland; 6 MRC Centre for Neuropsychiatric Genetics and Genomics, Neuroscience and Mental Health Research Institute, Cardiff University, Cardiff, United Kingdom; 7 Barts and The London Medical School, Queen Mary University of London, London, United Kingdom; 8 Department of Psychiatry, Neuropharmacology and Neurobiology Section, University of Birmingham, Birmingham, United Kingdom; 9 Department of Psychiatry, Trinity Centre for Health Science, Dublin, Ireland; 10 Department of Psychiatry, Washington University, St. Louis, Missouri, United States of America; 11 Department of Psychiatry, University of Bonn, Bonn, Germany; 12 Centre for Psychiatric Research, Aarhus University Hospital, Risskov, Denmark; 13 Division of Genetic Epidemiology in Psychiatry, Central Institute of Mental Health, Mannheim, Germany; 14 University Hospital Center and University of Lausanne, Lausanne, Switzerland; 15 GlaxoSmithKline Research & Development, Stockley Park, United Kingdom; 16 GlaxoSmithKline Research & Development, Verona, Italy; 17 Department of Psychiatry, University of Toronto, Toronto, Canada; 18 NeuroSearch A/S, Ballerup, Denmark; 19 NIHR Biomedical Research Centre for Mental Health, South London and Maudsley NHS Foundation Trust and Institute of Psychiatry, King's College London, London, United Kingdom; University of Muenster, Germany

## Abstract

**Background:**

Suicidal behaviour can be conceptualised as a continuum from suicidal ideation, to suicidal attempts to completed suicide. In this study we identify genes contributing to suicidal behaviour in the depression study RADIANT.

**Methodology/Principal Findings:**

A quantitative suicidality score was composed of two items from the SCAN interview. In addition, the 251 depression cases with a history of serious suicide attempts were classified to form a discrete trait. The quantitative trait was correlated with younger onset of depression and number of episodes of depression, but not with gender. A genome-wide association study of 2,023 depression cases was performed to identify genes that may contribute to suicidal behaviour. Two Munich depression studies were used as replication cohorts to test the most strongly associated SNPs. No SNP was associated at genome-wide significance level. For the quantitative trait, evidence of association was detected at *GFRA1*, a receptor for the neurotrophin GDRA (p = 2e-06). For the discrete trait of suicide attempt, SNPs in *KIAA1244 and RGS18* attained p-values of <5e-6. None of these SNPs showed evidence for replication in the additional cohorts tested. Candidate gene analysis provided some support for a polymorphism in *NTRK2*, which was previously associated with suicidality.

**Conclusions/Significance:**

This study provides a genome-wide assessment of possible genetic contribution to suicidal behaviour in depression but indicates a genetic architecture of multiple genes with small effects. Large cohorts will be required to dissect this further.

## Introduction

Suicide is a significant public health issue and a major cause of death, with the WHO estimating that suicide accounts for 1.5% of the deaths throughout the world. Attempted suicide is more frequent than completed suicide (lifetime prevalence of ∼3.5%), and approximately 10% of suicide attempters will commit suicide within 10 years [1]. Suicidal behaviour refers to the occurrence of suicide attempts that range from completed suicide, to highly lethal but failed suicide attempts, to suicide attempts of low lethality [2]. Suicidal ideation comprises suicidal thoughts or threats which may or may not be followed by action. Suicidality can be viewed as a continuum of increasing severity from suicidal ideation, to suicide attempts, to completed suicide [3]; [4]. Suicidal behaviour is strongly linked with psychiatric disorders, in particular, mood disorders and substance problems [5], with approximately 90% of suicide attempters having a psychiatric disorder.

Analyses from pooled twin studies of completed suicide showed a higher concordance in monozygotic than dizygotic twins (11% v. 2%), with an estimated heritability of completed suicide of approximately 43% (95% CI 27–60%), with no contribution from shared family environment [6]–[8]. Such studies of the familiality of completed suicide are limited by the small numbers, but indicate the existence of genes contributing to suicidal behaviour. Many family studies have shown an increase in suicidal behaviour, showing a high familiality of both attempted and completed suicide [9]. Such familial transmission of suicidal behaviour is not fully explained by co-morbid psychiatric disorders, and may be more closely related to aggression and impulsivity traits transmitted within the family [10]. Twin studies which extended the phenotype to suicidal ideation found some overlap of genetic contribution to suicidal ideation and suicidal attempts [8]. These studies together indicate that a broad spectrum of suicidal behaviour is likely to be partly under genetic control, and that part of the genetic contribution is independent of that for psychiatric disorder.

Although the existence of genetic vulnerability to suicidality is well-established, progress in the identification of its molecular basis has been slow. Functional candidate gene studies have identified few replicable associations and other candidate genes have been identified through expression studies [11]; [12]. More recently, a genome-wide association study on suicide attempt in mood disorder subjects found suggestive evidence for multiple loci that needs to be replicated [13]. The genetic contribution to treatment-emergent suicidal ideation (TESI) during antidepressant treatment has also been investigated [14], [15].

In this study, we investigate a broad phenotype of suicidal behaviour, a term we use here to encompass both suicidal ideation and suicidal attempts, in cases of major depressive disorder (MDD). Analyzing suicidal behaviour in a cohort of depression cases provides an *a priori* high risk group for suicidal behaviour that is appropriate for uncovering the genetic contribution to this complex phenotype. We defined a quantitative suicidality measure in depression cases from the RADIANT study, and established its correlation with other features of major depression. Through the use of suicidality as a continuum from ideation to suicide attempts as a quantitative trait, we aim to increase power to detect genes associated with suicidality. We also consider the trait of suicide attempt, which forms the upper tail of this quantitative trait. Since this is a retrospective study, with suicidality assessed by recall of the most severe episode of depression, the most severe end of spectrum (i.e. completed suicide) will be missing. To identify genes that underlie suicidality, we performed a GWAS and further investigated candidate genes previously implicated in susceptibility to suicidality. Regions showing strongest evidence for association were tested in two additional cohorts of depression cases from Munich (MARS, GSK-Munich) as a replication study.

## Results

We defined a continuous SCAN Suicidality (SSU) score using responses from two items in the SCAN questionnaire in the RADIANT studies, which comprise DeCC (cases ascertained in the UK), DeNt (cases ascertained across Europe) and a cohort from Bonn/Lausanne (ascertained in collaboration with GSK). SSU scores capture the distribution of suicidality in MDD cases from ideation to suicide attempt, and were available in a total of 2154 depression cases (2023 of which also met QC for genome-wide association) (Table 1). SSU scores were similar in DeCC (mean SSU score of 3.86), DeNt (mean SSU score = 3.87), and the Bonn/Lausanne samples (mean SSU = 3.57). The SSU score was symmetrically distributed, with 45.2% of cases having a score of 4. There was a high correlation between SSU scores in the subject's reported worst and second worst episode of depression, with 50.1% of cases having equal scores in both episodes, and 38.4% having a higher SSU score in the worst episode.

**Table 1:** Distribution of SSU score and study characteristics.

Study	No. individuals	Mean SSU score (s.d.)	No. with suicide attempts (%)	No. females (%)	Mean age onset, s.d. (years )	Mean age at interview, s.d. (years)
DeCC	1117	3.857 (1.639)	125 (11.3%)	767 (69.2%)	23.0 (11.6)	47.1 (12.0)
DeNt	898	3.867 (1.687)	125 (14.0%)	686 (77.1%)	21.9 (11.2)	44.9 (11.7)
Bonn/Lausanne	163	3.568 (1.652)	15 (9.7%)	115 (74.2%)	25.3 (12.8)	49.7 (12.5)
Total	2154	3.84 (1.661)	265 (12.3%)	1568 (72.8%)	22.7(11.5)	46.3 (12.0)

SSU score was significantly associated with the number of depressive episodes (p<2×10^−6^) and age at onset (p = 0.004), but not with sex (p = 0.72). The mean SSU score for cases with two episodes of depression was 3.7 compared to 3.9 for cases with three or more episodes (p = 0.007). Younger age of onset predicted higher SSU scores in the cases from the DeCC study (which were ascertained on the basis of recurrent depression only) (p = 9.7×10^−5^), but not in the DeNt study (p = 0.875), where cases additionally had a sibling with recurrent depression. These differences are not accounted by differences in mean ages of onset or SSU scores in these studies (Table 1).

There was a significant association between SSU score and Beck's Depression Inventory score (p<10^−8^), with higher BDI scores predicting higher SSU scores. Personality traits were assessed using the Eysenck Personality Questionnaire (EPQ) in all studies. There was a significant association of both EPQ-extroversion (EPQ-E) score (p<10^−8^), and EPQ-neuroticism (EPQ-N) (p<10^−8^) with SSU score. Individuals with higher EPQ-E scores had significantly lower SSU scores, whereas individuals with higher EPQ-N scores had significantly higher SSU scores.

The SCAN questions enable us to classify MDD cases as having made a serious attempt at suicide in their depressive episode. These cases lie in the upper tail of the SSU score, and 12.3% of depression cases were classified as having made a serious suicide attempt in the discrete SSU trait. The mean SSU score for the suicide attempters was 6.68, compared to 3.16 for the non-suicide attempters. These groups did not differ by sex, age at onset of depression or age at interview.

**Figure 1:**
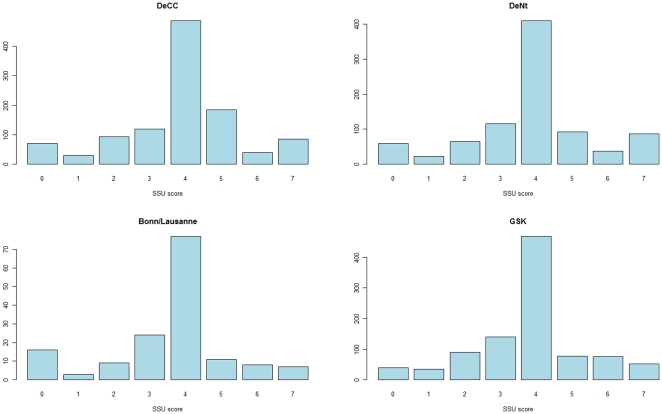
Distribution of SSU score, by study.

In the GSK-Munich replication cohort, SCAN information was available on 982 cases of recurrent depression. The distribution of SSU score was similar to RADIANT, with 47.8% of cases having a score of 4 (Figure 1). Similar correlations with clinical covariates were obtained as in RADIANT: SSU score was significantly correlated with age (p = 1.37e-05), but not with sex (p = 0.312). In total, 13.1% of 982 depression cases scored >5 on the SSU score and were therefore classified as making a suicide attempt. This figure is comparable with the 12.3% prevalence of suicide attempt in RADIANT, which had similar ascertainment criteria.

In the MARS replication cohort, 20.9% of 532 depression cases reported a suicide attempt; no quantitative trait information, equivalent to that from SCAN, was available. The prevalence of suicide attempt is substantially higher than in RADIANT or the GSK-Munich cohorts, which reflects the different definitions of lifetime suicide attempts (MARS) or suicidal behaviour in the two worst episodes of depression (RADIANT, GSK-Munich).

### Genome-wide association study

**Figure 2:**
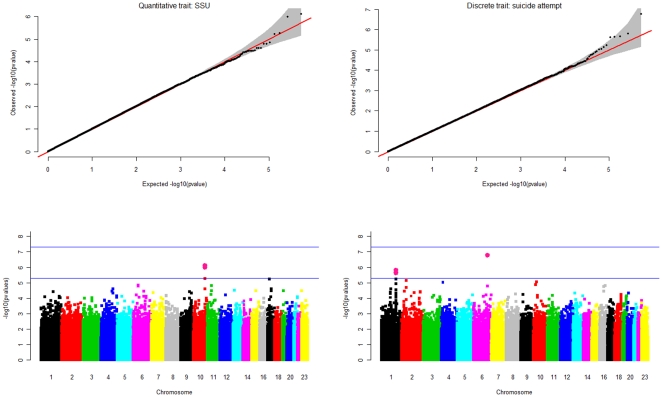
Summary of results for the quantitative suicidality trait (SSU) and the discrete trait of suicide attempt, showing quantile-quantile plots and Manhattan plots. Genomic control λ values were 1.007 (SSU score) and 1.012 (discrete trait).

No SNP was associated with suicidal behaviour at the genome-wide level of significance (p = 5×10^−8^) in the analysis of either the quantitative SSU score or the discrete trait of suicide attempt (Figure 2). Seven SNPs from three regions showed significance at our suggestive level of significance (p = 5×10^−6^; Table 2). In the analysis of quantitative SSU score, rs4751955 on chromosome 10 achieved a p-value of 7.57×10^−7^. This SNP is located in an intron of *GFRA1*, the GDNF-family receptor alpha 1 gene. The most significant result for the discrete trait of serious suicidal attempts was at rs203136 (p = 1.91×10^−7^) in gene *KIAA1244* on chromosome 6q23.3, which encodes the brefeldin A-inhibited guanine nucleotide-exchange protein 3 (BIG3). Four SNPs on chromosome 1 also reached suggestive significance in the analysis of the discrete trait (the most significant SNP is listed in Table 2), These SNPs lie in an 1.6 Mb gene desert between *RGS18* (which encodes a member of the regulator of G-protein signalling family) and *FAM5C* (family with sequence similarity 5, member C). The most significant results in the quantitative and discrete SSU score traits did not occur at the same SNPs, but there was strong consistency between results overall, as expected since the discrete SSU score is defined by the upper tail of the quantitative distribution. A p-value of <0.05 was attained at 27,186 SNPs in the quantitative analysis, and 27,852 SNPs with the discrete trait. Of these, 7707 (28%) were common to both analyses, and all SNPs had effect sizes in the same direction.

**Table 2:** Most significantly associated SNPs for quantitative SSU score and discrete SSU trait, showing only top SNP from each genomic region.

					RADIANT			GSK		MARS		Meta-analysis		
SNP	CHR	Basepair position	Tested allele	Allele freq.	Beta/OR	SE	RADIANT p-value	Beta/OR	p-value	Beta/OR	p-value	Beta/OR	p-value	Closest Gene
**Quantitative trait: SSU score**														
rs4751955	10	117,913,215	A	0.45	0.268	0.054	7.75E-07	0.027	0.705	-	-	0.180	2.84E-05	*GFRA1 (intronic)*
rs12948266	17	15,362,260	C	0.14	−0.349	0.077	6.00E-06	−0.042	0.681	-	-	−0.238	0.000107	*FAM18B2 (intronic)*
rs510153	6	33,678,053	A	0.23	−0.280	0.064	1.42E-05	0.045	0.581	-	-	−0.155	0.002181	*FLJ43752/BAK1*
rs1532096	11	42,869,929	G	0.14	−0.329	0.076	1.61E-05	0.052	0.604	-	-	−0.189	0.001785	*LOC399881 (370 kb)*
rs17023187	4	148,542,379	C	0.11	−0.361	0.086	2.53E-05	0.073	0.522	-	-	−0.206	0.00269	*EDNRA (80kb)*
rs1881140	17	45,671,871	G	0.46	−0.220	0.052	2.53E-05	0.024	0.739	-	-	−0.135	0.001315	*LOC729160 (34 kb)*
rs11841788	13	30,888,519	G	0.14	0.315	0.075	3.14E-05	−0.054	0.590	-	-	0.182	0.002545	*B3GALTL (85 kb)*
rs1882411	23	5,982,382	T	0.46	0.237	0.057	3.35E-05	-	-	-	-	-	-	*NLGN4X (25 kb)*
rs3803414	15	63,993,258	A	0.09	−0.383	0.092	3.40E-05	−0.045	0.714	-	-	−0.263	0.00038	*MEGF11 (coding)*
rs8101893	19	14,801,001	C	0.28	0.242	0.058	3.41E-05	0.075	0.370	-	-	0.188	8.72E-05	*OR7A5 (1 kb)*
**Discrete trait: Suicide attempt**														
rs203136	6	138,647,945	G	0.36	1.662	0.097	1.74E-07	1.102	0.503	0.960	0.783	1.328	6.24E-05	*KIAA1244 (intronic)*
rs12751302	1	189,813,197	T	0.34	0.584	0.112	1.61E-06	-	-	0.999	0.993	0.707	0.000116	*RGS18/FAM5C*
rs4953249	2	45,846,361	G	0.10	1.865	0.139	7.30E-06	0.731	0.178	0.902	0.686	1.337	0.007151	*PRKCE (intronic)*
rs703088	10	21,180,261	A	0.05	2.178	0.175	9.01E-06	1.092	0.734	0.989	0.970	1.572	0.000537	*NEBL (intronic)*
rs17387100	4	15,604,223	G	0.08	1.925	0.148	9.53E-06	0.932	0.782	0.614	0.123	1.401	0.004519	*PROM1 (intronic)*
rs7904694	10	14,013,585	T	0.05	2.198	0.181	1.34E-05	1.558	0.091	0.729	0.373	1.695	0.000121	*FRMD4A (intronic)*
rs16972539	16	72,362,202	T	0.14	1.707	0.124	1.56E-05	1.090	0.635	1.275	0.260	1.440	7.77E-05	*LOC441506 (170 kb)*
rs8061077	16	57,255,287	T	0.22	1.590	0.108	1.80E-05	0.811	0.201	1.255	0.187	1.288	0.001572	*FLJ10815 (2.5 kb)*
rs6583045	1	108,088,494	A	0.40	1.504	0.097	2.57E-05	0.924	0.559	0.945	0.713	1.197	0.01017	*VAV3 (4.6 kb)*
rs2305450	2	29,236,878	G	0.09	1.808	0.144	3.69E-05	0.770	0.277	0.657	0.148	1.281	0.02904	*CLIP4 (intronic)*

In the two Munich replication cohorts, no evidence for association was found at the top SNPs in the RADIANT study for either the quantitative trait of suicidal behaviour (GSK-Munich study) or the discrete suicide attempt trait (GSK-Munich, MARS) (Table 2). One other genome-wide study of suicide attempts in MDD has been published, using cases from the STAR*D trial (13). We performed a meta-analysis of the strongest results from that study and RADIANT. The SNPs showed no consistent replication in RADIANT, with the strongest evidence for association (p = 0.0010) arising at rs1377287, located in an intron of the solute carrier family 4, member 4 gene, *SLC4A4* (Table 3). Although the OR estimates for Munich studies MARS and GSK are in the same direction at the RADIANT effect at rs1377287 (OR = 0.823, 0.861), the p-values add little support to the results, and a meta-analysis across studies decreased in significance (Fisher's method meta-analysis p-value = 6×10^−5^).

**Table 3:** Comparison of most significant results from GWAS of suicide attempts in STAR*D [13], showing p-values from RADIANT study, and meta-analysis p-values using Fisher's method.

				STAR*D			RADIANT			
SNP	CHR	Base pair position	Closest Gene	Alleles	OR	p-value	Tested allele	OR	p-value	Fisher's method p-value
rs1377287	4	72355439	*SLC4A4*	AG	0.536	0.00030	G	0.556	0.0010	4.870E-06
rs276420	13	39470070	*COG6*	GT	0.666	0.00043	T	0.759	0.0048	2.956E-05
rs7339164	13	39470053	*COG6*	AG	1.567	0.00013	A	0.763	0.0081	1.541E-05
rs3734662	6	90707807	*BACH2*	CT	0.664	0.00083	C	0.779	0.0110	1.149E-04
rs9603665	13	39500347	*COG6*	CT	0.680	0.00091	T	0.772	0.0113	1.287E-04
rs1777077	14	42258660	*LRFN5*	CT	1.628	0.00085	C	1.303	0.0165	1.704E-04
rs7941624	11	80658536	*MGC33846*	AG	1.830	0.00077	G	0.739	0.0173	1.620E-04
rs6966472	7	84537323	*SEMA3D*	CT	1.834	0.00019	C	1.358	0.0189	4.754E-05
rs10897779	11	80661868	*MGC33846*	GT	1.661	0.00099	T	0.753	0.0222	2.573E-04
rs10890590	11	106171269	*GUCY1A2*	AG	0.573	0.00046	A	0.777	0.0257	1.460E-04
rs17459015	6	104924468	*HACE1*	CT	1.636	0.00051	C	1.292	0.0263	1.631E-04
rs2240394	7	84530877	*SEMA3D*	CT	1.718	0.00044	C	1.332	0.0282	1.516E-04
rs1409470	13	104284951	*DAOA*	AG	0.643	0.00026	G	1.227	0.0340	1.127E-04
rs270678	6	104891512	*HACE1*	CT	1.645	0.00045	C	1.274	0.0356	1.943E-04

### Candidate gene analyses

We tested for association with a set of 33 candidate genes (875 SNPs) previously implicated in susceptibility to suicidality through their function or previous genetic association studies. No strongly significant results were obtained for SNPs in any candidate gene. Table 4 lists the genes in which at least one SNP achieved p-value<0.01 in the quantitative or discrete SSU trait, and a correction for multiple testing of SNPs within the gene (only the most significant SNP for each gene is shown). None of these findings would survive an additional correction for multiple testing across the number of genes analysed. Three SNPs achieved nominal significance: *HTR1A* in the trait of suicide attempts, *CCK* and *RSG18* in the association with quantitative SSU trait, but none of these were associated in the Munich cohorts. Two SNPs, in *NTRK2* and *SCN8A* achieve nominally significant p-values (<0.05) in Munich cohorts, but with opposite direction of association to RADIANT. The *NTRK2* SNP, rs10868235, associated with suicide attempts in the German depression cases and has already been reported in the Munich cohorts [16].

**Table 4:** Candidate gene analysis for quantitative and discrete SSU trait, listing SNPs within gene (or in 20-kb flanking regions) achieving p<0.01.

Gene	CHR	SNP	Basepair position	Tested allele	Beta/OR	Number of SNPs in gene	RADIANT p-value	M_eff	RADIANT p-value (corrected)	GSK Beta/OR	GSK p-value	MARS Beta/OR	MARS p-value
Quantitative trait: SSU score													
*CCK*	3	rs10460960	42,283,739	G	0.272	12	0.0016	8	0.013	0.120	0.281	-	-
*RGS2*	1	rs16829458	191,034,947	A	−0.310	4	0.0019	3.6	0.007	−0.018	0.893	-	-
*NTRK2*	9	rs6559838	86,743,383	T	−0.197	95	0.0021	43.6	0.090	−0.039	0.599	-	-
*GRIA3*	X	rs4825847	122,215,515	C	−0.184	60	0.0023	36.6	0.085	-	-	-	-
*HTR2A*	13	rs4942577	46,295,455	C	0.171	51	0.0026	26.9	0.070	−0.023	0.761	-	-
*IL28RA*	1	rs12028945	24,354,591	A	0.235	25	0.0040	18.3	0.074	-	-	-	-
*FKPB5*	6	rs2766545	35,821,009	A	−0.147	22	0.0049	11.3	0.055	-	-	-	-
*SCN8A*	12	rs12424271	50,301,235	A	−0.217	29	0.0054	13	0.070	0.253	0.026	-	-
*WSF1*	4	rs7655674	6,366,993	T	−0.203	22	0.0099	11	0.109	−0.050	0.658	-	-
Discrete trait: Suicide attempt													
*NTRK2*	9	rs10868235	86,683,575	C	0.738	95	0.0015	36.6	0.056	1.405	0.012	1.008	0.955
*CRHR2*	7	rs2284218	30,680,858	C	0.756	21	0.0059	13.1	0.078	1.078	0.570	1.237	0.154
*IL28RA*	1	rs12028945	24,354,591	A	1.430	25	0.0084	8	0.067	-	-	0.718	0.199
*HTR1A*	5	rs1364043	63,286,607	G	0.727	2	0.0087	1.7	0.015	0.944	0.716	1.140	0.455
*NOS_1*	12	rs4766834	116,124,928	A	1.286	50	0.0089	24.4	0.216	1.114	0.399	1.208	0.198

M_eff_ = effective number of tests in a gene. Only the most significant SNP in each gene is listed.

## Discussion

We carried out a GWAS of suicidality in 2023 subjects with DSM-IV and/or ICD-10 diagnosis of MDD in the RADIANT studies. No genome-wide evidence of association was detected for either suicidality measure analysed. SNPs in three genetic regions reached our threshold for suggestive evidence of association: in *GFRA1*, when analysing suicidality as a quantitative trait and two regions (in *KIAA1244*, and between *RGS18* and *FAM5C*) when analysing the discrete trait of suicide attempt. *GFRA1* is a receptor of the neurotrophin GDNF which has been widely investigated in mood disorders (MDD and bipolar disorder), schizophrenia and treatment responses [17]; [18]; [19]; [20]). Another GDNF receptor gene, *GFRA2*, has been associated with antipsychotic response in a GWAS [21]. Given BDNF's reported involvement in suicidal behaviour [22]; [23], these new results implicating GDNF and its receptor suggest that neurotrophic systems play a role in suicidal behaviour, possibly through an inability of neuronal systems to exhibit appropriate adaptive plasticity.

The location of the strongest association signal within a gene of high potential relevance to psychiatric disorders (*GFRA1*) increases the probability that these findings may be true but modest signals of association. However, none of the SNPs showing evidence for association in the RADIANT study were significantly associated with SSU or with suicide attempt in the Munich GSK and MARS studies. This lack of replication is not unsurprising in a complex phenotype such as suicidal behaviour where the genetic contribution is likely comprise small effects. A recent meta-analysis of genetic association with suicide attempts in bipolar disorder and MDD also failed to show replication across the different studies included [13]. In the STAR*D depression study, they showed genome-wide significance for SNPs in *AB13BP*, which did not replicate in their replication cohort of the NESTA/NTR. Meta-analysis of SNPs associated with suicide attempts in STAR*D with results from RADIANT failed to identify SNPs with suggestive evidence of association. Meta-analysis across large samples with homogeneous definitions of suicide behaviour and, possibly, underlying psychiatric disorder will be required for further dissection of the genetic contribution to suicidality.

In our analysis of candidate genes for suicidal behaviour, three of the top associations survived a correction for multiple testing of SNPs within the gene, but fell short of the required significance for multiple testing across genes. For *HTR1A*, our associated SNP is in low linkage disequilibrium with a SNP, rs6295, previous suggested to be associated with suicide attempt [24]. In *RGS2*, the associated SNP in our analysis was not in linkage disequilibrium with the two SNPs reported as associated with suicidality in a Japanese population [25]. Similarly, in *CCK* (cholecystokinin) an association was observed at −196G/A in male Japanese suicide attempters, which has not been replicated further [26]. LD between this variant and our associated SNP is unknown.

The current study has several limitations. First of all, the SCAN SSU score was defined from two items of the depression section of the SCAN interview. Neither the instrument nor our depression studies were designed primarily to address suicidality. The validity of the score assumes a consistency across the two SCAN items used in its definition: i.e. that an increase of one score point in the question on suicidal ideation is equivalent to an increase in one score point in suicidal action, and that within each question, the responses have a linear relationship with increasing severity of suicidality. Our sample of MDD cases had good power to detect genes of moderate effect size, but had limited power to identify genes of small effect size that are assumed to be involved in suicidal behaviour. Therefore, it is likely that the reported findings are false positives, as implied by the lack of replication in the two additional cohorts. Our investigation was performed within a single disorder (major depression) which could be seen as a limitation of this study. However, focusing on suicidal behaviour within a single disorder allows the distinction between genes relating to suicide *per se* from those associated with the disorder itself (major depression) [15], [27].

In conclusion, we have performed a GWAS for suicidality encompassing both ideation and behaviour in MDD. We failed to detect evidence for association at a genome-wide level of significance, and the strongest results in our study were not replicated in analysis of independent MDD cohorts with a similar assessment of suicidal behaviour. Further attempts to replicate these findings will be necessary to determine whether the suggestive signals detected in this study are true effects or false positives.

## Materials and Methods

### Ethics Statement

All participants in this study gave written informed consent. The study was approved by the local Ethics Committee at the Institute of Psychiatry, King's College London.

### Samples

MDD cases from the RADIANT studies DeCC and DeNt with information on suicidal ideation and suicide attempts while in a depressive episode were analysed. The DeCC (Depression Case Control) sample consists of 1346 cases (69.3% women) of recurrent depression fulfilling DSM-IV and/or ICD-10 criteria of at least moderate severity ascertained from three UK clinical sites (London, Cardiff and Birmingham) [28]. The mean age of onset was 22.9 years (SD 10.8 years). Subjects were identified from psychiatric clinics, hospitals, general medical practices, and from volunteers responding to media advertisements. Retrospective information on the subject's two most severe episodes of depression was collected using Schedules for Clinical Assessment in Neuropsychiatry SCAN [29], which includes two questions on suicidal ideation and behavior.

The DeNt (Depression Network) affected sibling pair linkage study [30], [31] comprises cases of recurrent depression of at least moderate severity. Subjects were ascertained from three UK sites (London, Cardiff and Birmingham), four other European sites (Aarhus, Bonn, Dublin and Lausanne) and a site in St. Louis, USA. Only the proband from each family, for whom genome-wide genotypes were available, was included in the phenotype and genotype analysis presented here (n = 898). As in DeCC, symptom type and severity for the subject's worst and second-worst episode of depression were assessed using the SCAN interview. An additional 163 cases of recurrent depression collected in Bonn and Lausanne, using exactly the same protocol as the DeNt study, were also analysed.

All cases from all studies fulfilled DSM-IV and/or ICD-10 criteria of at least moderate severity. Study co-ordinators for all studies were trained by A.E.F. ensuring homogeneity of clinical data collected. Exclusion criteria across studies were broadly comparable. Subjects were excluded if there was a history or family history of schizophrenia or bipolar disorder, for mood-incongruent psychosis, or if mood symptoms were related to alcohol or substance misuse. All study participants completed the Beck's Depression Inventory (BDI) and the Eysenck Personality Questionnaire (EPQ).

### Suicidality phenotype

A new variable named **S**CAN **SU**cidality (SSU) score was created in RADIANT. This measure combines responses to two SCAN items: 6.012, which assesses *tedium vitae*, and 6.011, which rates suicide attempt and self-harm during the episode of depression. In 6.011, a score for suicide attempt and self-harm between 0 and 4 is assigned, on the scale 0: absent; 1: deliberately considered suicide or self-injury but made no attempt; 2: injured self or made an attempt but no serious harm results; 3; as 2. but with serious self-harm; 4: made an attempt at suicide designed to result in death. Question 6.012, which is only included when the response to 6.011 score is 0, is scored at between 0 and 3, assessing response to the question ‘Have you felt that life was not worth living or that you would not care if you didn't wake in the morning?’ For a non-zero score on 6.011, the SSU score was defined equal to the 6.011-score+3 (giving integer scores of 4, 5, 6, 7). Where the 6.011 score was zero, the SSU score was defined equal to the 6.012 (*tedium vitae*) score, giving values of 0 to 3. The SSU algorithm allows action (6.011) to “trump” ideation (6.012), and gives a distribution of integer-valued scores of between 0 and 7. SCAN information for both the worst and second-worst episode of depression were analysed, and the maximum SSU score was used.

Serious suicidal attempts were defined as an SSU score of 6 or more, corresponding to a suicide attempt “with serious harm” or “an attempt at suicide designed to result in death” (that is, a score of 3 or 4 on SCAN item 6.011).

### Genotyping

DNA was extracted as described previously. Concentrations of all samples were adjusted to 50 ng/µl and 15 µl of each robotically dispensed into barcoded 96-well plates. Concentration, fragmentation and response to PCR were determined. Whole-genome genotyping was performed using the Illumina HumanHap610-Quad BeadChip by the Centre National de Génotypage (CNG), France. All DNA samples were subjected to stringent quality control, and processing was carried out under full LIMS control. The raw data were analysed using GTS Image and extracted for statistical analysis.

### Quality control

Stringent quality control procedures were applied to individual and SNP data. Individuals were excluded if their genotypic data showed missing rate >1%, abnormal heterozygosity, a sex assignment that conflicted with phenotypic data, if they were related (up to 2^nd^ degree) with other study members, or of non-European ancestry. Non-European ancestry was determined using principal components analysis of HapMap CEU, JPT, CHB, YRI and GIH populations with EIGENSTRAT [32]. Related or duplicate cases were identified through identity-by-state sharing analysis; for each pair related up to second degree relationships, the individual with lower genotyping completeness was omitted. SNPs with minor allele frequency <1% or showing departure from Hardy-Weinberg equilibrium (p<1×10^−5^) were excluded. EIGENSTRAT analysis was performed again after QC procedures, and five ancestry-informative principal components (PCs) were used as covariates in association testing. For further details of quality control see Lewis et al. [33]. The final data set comprised 2023 depression cases with quantitative SSU scores (including 251 cases with suicide attempt) which were genotyped on 532,774 SNPs.

### Statistical analysis

The relationship between the quantitative SSU trait and relevant covariates (study, sex, age of onset, number of depressive episodes) was determined using linear regression and binomial tests. The quantitative SSU score (which lies on an ordinal scale of 0–7) was treated as a continuous variable. In the genetic analysis, association between SNPs and suicidality in MDD was tested using linear regression for the quantitative SSU score and logistic regression for the discrete trait, assuming a log-additive model for SNP genotype. In all analyses, five ancestry-informative principal components were included as covariates. The genomic control parameter λ was calculated for each analysis to assess test statistic inflation due to residual population stratification [34]. Genomic control λ values were 1.007 (SSU score) and 1.012 (for the discrete trait), indicating no inflation of test statistics from uncorrected population stratification or other systematic bias. Analyses were implemented using PLINK 1.07 [35] and R (www.r-project.org).

Two thresholds of significance were used to interpret association results: genome-wide evidence for association at a p-value threshold of 5×10^−8^ [36] and suggestive evidence of association, set two orders of magnitude lower, at p<5×10^−6^. These thresholds provide appropriate correction for multiple testing of SNPs across the genome; no additional correction was made for analysis of two phenotypes. The power of the study to detect association was calculated assuming a continuous quantitative trait, using Genetic Power Calculator (GPC, [37]). For a SNP accounting for 1.5% of additive genetic variance, 2023 individuals gives approximately 83% power to detect association at the suggestive level of significance, and 53% power at genome-wide level of significance. For the discrete trait (251 suicide attempt cases, 1772 controls), this study had 85% power at suggestive level of significance (and 56% power at genome-wide significance) to detect a SNP of frequency 0.3 conferring a genotype relative risk of 1.6 under a log-additive model.

We also tested specifically for association with a set of 33 candidate genes previously implicated in susceptibility to suicidality through their function or previous genetic association studies (Table S1) [14]. In total, 875 SNPs lying in the genes or in 20 kb flanking regions were tested. Multiple testing correction for SNPs within genes was used the web-based SNPSpD software (http://gump.qimr.edu.au/general/daleN/SNPSpD) which estimates the number of independent tests (M*_eff_*), accounting for LD between genotyped SNPs [38]; [39]. A gene-wide threshold for significance was then calculated as α_corr_ = 0.05/M*_eff_*. No correction for multiple testing across genes was applied.

### Replication study

Replication of the top SNPs associated with the SSU score and suicide attempt discrete traits was performed in two MDD cohorts from Germany. The GSK-Munich cohort [40] was collected using identical measurement instruments to the RADIANT study, with investigators trained by A.E.F. This cohort comprises 982 cases with information on the SCAN and genoytped on the Illumina 550K platform. From the Munich Antidepressant Response Signature (MARS) project, 549 cases of depression genotyped on the Illumina 610K platform were available [41] [42]. Lifetime history of suicide attempt was determined in a semi-structured clinical interview as part of the MARS study and from the suicide item on the Hamilton Scale for Depression rating scale. Standard genotype QC procedures were applied, and no correction was necessary for population stratification. Analysis of the quantitative SSU score (GSK-Munich only) and the discrete suicide attempt variable (both cohorts) was performed as in the RADIANT study. Meta-analysis of top hits from RADIANT was performed using PLINK under a fixed effects model. We also tested for replication to SNPs that were associated with suicide attempt in 1,273 depression cases in the STAR*D study [43]. Meta-analysis of SNPs with association p-values of <0.001 (listed in Supplementary Table 2 of Perlis et al. [13]) which were also genotyped in our studies (n = 323) was performed using Fisher's method for combining p-values.

## Supporting Information

Table S1 **List of candidate genes and their rationale for their inclusion.**
(DOCX)

## References

[1] Suominen K, Isometsa E, Suokas J, Haukka J, Achte K (2004). Completed suicide after a suicide attempt: a 37-year follow-up study.. Am J Psychiatry.

[2] Mann JJ (2002). A current perspective of suicide and attempted suicide.. Ann Intern Med.

[3] Courtet P, Jollant F, Castelnau D, Buresi C, Malafosse A (2005). Suicidal behavior: relationship between phenotype and serotonergic genotype.. Am J Med Genet C Semin Med Genet.

[4] Perroud N, Uher R, Hauser J, Rietschel M, Henigsberg N (2010). History of suicide attempts among patients with depression in the GENDEP project.. J Affect Disord.

[5] Arsenault-Lapierre G, Kim C, Turecki G (2004). Psychiatric diagnoses in 3275 suicides: A meta-analysis.. BMC Psychiatry.

[6] Roy A, Segal NL, Centerwall BS, Robinette CD (1991). Suicide in twins.. Arch Gen Psychiatry.

[7] McGuffin P, Marusic A, Farmer A (2001). What can psychiatric genetics offer suicidology?. Crisis.

[8] Fu Q, Heath AC, Bucholz KK, Nelson EC, Glowinski AL (2002). A twin study of genetic and environmental influences on suicidality in men.. Psychol Med.

[9] Brent DA, Mann JJ (2005). Family genetic studies, suicide, and suicidal behavior.. Am J Med Genet C Semin Med Genet.

[10] Turecki G (2005). Dissecting the suicide phenotype: the role of impulsive-aggressive behaviours.. J Psychiatry Neurosci.

[11] Brezo J, Klempan T, Turecki G (2008). The genetics of suicide: a critical review of molecular studies.. Psychiatr Clin North Am.

[12] Bondy B, Buettner A, Zill P (2006). Genetics of suicide.. Mol Psychiatry.

[13] Perlis RH, Huang J, Purcell S, Fava M, Rush AJ (2010). Genome-Wide Association Study of Suicide Attempts in Mood Disorder Patients.. Am J Psychiatry.

[14] Perroud N, Uher R, Ng MY, Guipponi M, Hauser J (2010). Genome-wide association study of increasing suicidal ideation during antidepressant treatment in the GENDEP project.. Pharmacogenomics J.

[15] Laje G, Allen AS, Akula N, Manji H, John Rush A (2009). Genome-wide association study of suicidal ideation emerging during citalopram treatment of depressed outpatients.. Pharmacogenet Genomics.

[16] Kohli MA, Salyakina D, Pfennig A, Lucae S, Horstmann S (2010). Association of Genetic Variants in the Neurotrophic Receptor-Encoding Gene NTRK2 and a Lifetime History of Suicide Attempts in Depressed Patients.. Arch Gen Psychiatry.

[17] Rosa AR, Frey BN, Andreazza AC, Cereser KM, Cunha AB (2006). Increased serum glial cell line-derived neurotrophic factor immunocontent during manic and depressive episodes in individuals with bipolar disorder.. Neurosci Lett.

[18] Chen PS, Peng GS, Li G, Yang S, Wu X (2006). Valproate protects dopaminergic neurons in midbrain neuron/glia cultures by stimulating the release of neurotrophic factors from astrocytes.. Mol Psychiatry.

[19] Otsuki K, Uchida S, Watanuki T, Wakabayashi Y, Fujimoto M (2008). Altered expression of neurotrophic factors in patients with major depression.. J Psychiatr Res.

[20] Michel TM, Frangou S, Camara S, Thiemeyer D, Jecel J (2008). Altered glial cell line-derived neurotrophic factor (GDNF) concentrations in the brain of patients with depressive disorder: a comparative post-mortem study.. Eur Psychiatry.

[21] Lavedan C, Licamele L, Volpi S, Hamilton J, Heaton C (2009). Association of the NPAS3 gene and five other loci with response to the antipsychotic iloperidone identified in a whole genome association study.. Mol Psychiatry.

[22] Dwivedi Y, Rizavi HS, Conley RR, Roberts RC, Tamminga CA (2003). Altered gene expression of brain-derived neurotrophic factor and receptor tyrosine kinase B in postmortem brain of suicide subjects.. Arch Gen Psychiatry.

[23] Kim B, Kim CY, Hong JP, Kim SY, Lee C (2008). Brain-derived neurotrophic factor Val/Met polymorphism and bipolar disorder. Association of the Met allele with suicidal behavior of bipolar patients.. Neuropsychobiology.

[24] Wasserman D, Geijer T, Sokolowski M, Rozanov V, Wasserman J (2006). The serotonin 1A receptor C(-1019)G polymorphism in relation to suicide attempt.. Behav Brain Funct.

[25] Cui H, Nishiguchi N, Ivleva E, Yanagi M, Fukutake M (2008). Association of RGS2 gene polymorphisms with suicide and increased RGS2 immunoreactivity in the postmortem brain of suicide victims.. Neuropsychopharmacology.

[26] Shindo S, Yoshioka N (2005). Polymorphisms of the cholecystokinin gene promoter region in suicide victims in Japan.. Forensic Sci Int.

[27] Uher R, Perroud N (2010). Probing the genome to understand suicide.. Am J Psychiatry.

[28] Cohen-Woods S, Gaysina D, Craddock N, Farmer A, Gray J (2009). Depression Case Control (DeCC) Study fails to support involvement of the muscarinic acetylcholine receptor M2 (CHRM2) gene in recurrent major depressive disorder.. Hum Mol Genet.

[29] Wing JK, Babor T, Brugha T, Burke J, Cooper JE (1990). SCAN. Schedules for Clinical Assessment in Neuropsychiatry.. Arch Gen Psychiatry.

[30] Farmer A, Breen G, Brewster S, Craddock N, Gill M (2004). The Depression Network (DeNT) Study: methodology and sociodemographic characteristics of the first 470 affected sibling pairs from a large multi-site linkage genetic study.. BMC Psychiatry.

[31] McGuffin P, Knight J, Breen G, Brewster S, Boyd PR (2005). Whole genome linkage scan of recurrent depressive disorder from the depression network study.. Hum Mol Genet.

[32] Price AL, Patterson NJ, Plenge RM, Weinblatt ME, Shadick NA (2006). Principal components analysis corrects for stratification in genome-wide association studies.. Nat Genet.

[33] Lewis CM, Ng MY, Butler AW, Cohen-Woods S, Uher R (2010). Genome-wide association study of major recurrent depression in the U.K. population.. Am J Psychiatry.

[34] Devlin B, Roeder K (1999). Genomic control for association studies.. Biometrics.

[35] Purcell S, Neale B, Todd-Brown K, Thomas L, Ferreira MA (2007). PLINK: a tool set for whole-genome association and population-based linkage analyses.. Am J Hum Genet.

[36] Dudbridge F, Gusnanto A (2008). Estimation of significance thresholds for genomewide association scans.. Genet Epidemiol.

[37] Purcell S, Cherny SS, Sham PC (2003). Genetic Power Calculator: design of linkage and association genetic mapping studies of complex traits.. Bioinformatics.

[38] Nyholt DR (2004). A simple correction for multiple testing for single-nucleotide polymorphisms in linkage disequilibrium with each other.. Am J Hum Genet.

[39] Li J, Ji L (2005). Adjusting multiple testing in multilocus analyses using the eigenvalues of a correlation matrix.. Heredity.

[40] Muglia P, Tozzi F, Galwey NW, Francks C, Upmanyu R (2010). Genome-wide association study of recurrent major depressive disorder in two European case-control cohorts.. Mol Psychiatry.

[41] Kunzel HE, Binder EB, Nickel T, Ising M, Fuchs B (2003). Pharmacological and nonpharmacological factors influencing hypothalamic-pituitary-adrenocortical axis reactivity in acutely depressed psychiatric in-patients, measured by the Dex-CRH test.. Neuropsychopharmacology.

[42] Binder EB, Salyakina D, Lichtner P, Wochnik GM, Ising M (2004). Polymorphisms in FKBP5 are associated with increased recurrence of depressive episodes and rapid response to antidepressant treatment.. Nat Genet.

[43] Rush AJ, Fava M, Wisniewski SR, Lavori PW, Trivedi MH (2004). Sequenced treatment alternatives to relieve depression (STAR*D): rationale and design.. Control Clin Trials.

