# Supplementary material for: Genomewide Association Scan of Suicidal Thoughts and Behaviour in Major Depression
Source: PLoS One. 2011 Jul 5;6(7):e20690. doi: 10.1371/journal.pone.0020690 (PMC3130038; doi:10.1371/journal.pone.0020690)
Supplement: Table S1 — List of candidate genes and their rationale for their inclusion. (DOCX) [file pone.0020690.s001.docx]

**Table S1**: List of candidate genes and their rationale for their inclusion. For dopamine, norepinephrine, serotonin and monoamine signaling genes, only those previously involved in suicidal behaviors have been considered as candidate genes. Candidate genes were based on a review paper unless stated otherwise.

| **Gene symbol** | **Description** | **Rationale** |
| --- | --- | --- |
| *DRD2* | dopamine receptor D2 | Dopamine signaling |
| *ADRA2A* | adrenergic alpha-2A receptor | Norepinephrine signaling |
| *TH* | tyrosine hydroxylase | Norepinephrine and Dopamine signaling |
| *COMT* | catechol-O-methyltransferase | Monoamine signaling |
| *MAOA* | monoamine oxydase A | Monoamine signaling |
| *HTR1A* | serotonin receptor 1A | Serotonin signaling |
| *HTR2A* | serotonin receptor 2A | Serotonin signaling |
| *HTR1B* | serotonin receptor 1B | Serotonin signaling |
| *SLC6A4* | serotonin transporter | Serotonin signaling |
| *TPH1* | tryptophan hydoxylase 1 | Serotonin signaling |
| *TPH2* | tryptophan hydoxylase 2 | Serotonin signaling |
| *BDNF* | Brain-derived neurotrophic factor | Reported association with suicidal ideation during antidepressant treatment |
| *NTRK2* | neurotrophic tyrosine kinase, receptor, type 2 | BDNF receptor |
| *CREB1* | cyclic AMP response element binding protein | Reported association with suicidal ideation during antidepressant treatment |
| *GRIA3* | glutamate receptor 3 | Reported association with suicidal ideation during antidepressant treatment |
| *GRIK2* | glutamate receptor 6 | Reported association with suicidal ideation during antidepressant treatment |
| *IL28RA* | interleukin 28 receptor | Reported association with suicidal ideation during antidepressant treatment |
| *PAPLN* | papilin | Reported association with suicidal ideation during antidepressant treatment |
| *FKBP5* | FK506-binding protein 5 | Reported association with suicidal ideation during antidepressant treatment |
| *CCK* | cholecystokinin | Reported association with suicide attempts |
| *CRHR2* | corticotropin-releasing hormone receptor 2 | Reported association with suicide attempts |
| *DHCR7* | 7-dehydrocholesterol reductase | Reported association with suicide attempts |
| *NOS1* | nitric oxide synthase 1 | Reported association with suicide attempts |
| *RABAC1* | prenylated Rab acceptor 1 | Reported association with suicide attempts |
| *SCN8A* | sodium channel, voltage gated, type VIII, alpha polypeptide | Reported association with suicide attempts |
| *TBX19* | hypothalamic-pituitary-adrenocortical axis regulatory factor | Reported association with suicide attempts |
| *VAMP4* | vesicle-associated membrane protein 4 | Reported association with suicide attempts |
| *ACE* | angiotensin-converting enzyme | Reported association with suicide |
| *NOS3* | nitric oxide synthase 3 | Reported association with suicide |
| *RGS2* | regulator of G-protein signaling 2 | Reported association with suicide |
| *SAT1* | spermidine/spermine N1-acetyltransferase | Reported association with suicide |
|
| *WSF1* | Wolfram syndrome 1 | Reported association with suicide |
| *YWHAE* | tyrosine 3-monooxygenase/tryptophan 5-monooxygenase activation protein, epsilon polypeptide | Reported association with suicide |

**References**

1. Brezo J, Klempan T, Turecki G (2008) The genetics of suicide: a critical review of molecular studies. Psychiatr Clin North Am 31: 179-203.

2. Perroud N, Aitchison KJ, Uher R, Smith R, Huezo-Diaz P, et al. (2009) Genetic predictors of increase in suicidal ideation during antidepressant treatment in the GENDEP project. Neuropsychopharmacology 34: 2517-2528.

3. Laje G, Paddock S, Manji H, Rush AJ, Wilson AF, et al. (2007) Genetic markers of suicidal ideation emerging during citalopram treatment of major depression. Am J Psychiatry 164: 1530-1538.

4. Laje G, Allen AS, Akula N, Manji H, John Rush A, et al. (2009) Genome-wide association study of suicidal ideation emerging during citalopram treatment of depressed outpatients. Pharmacogenet Genomics 19: 666-674.

5. Brent D, Melhem N, Ferrell R, Emslie G, Wagner KD, et al. (2009) Association of FKBP5 Polymorphisms With Suicidal Events in the Treatment of Resistant Depression in Adolescents (TORDIA) Study. Am J Psychiatry.

6. Guipponi M, Deutsch S, Kohler K, Perroud N, Le Gal F, et al. (2009) Genetic and epigenetic analysis of SSAT gene dysregulation in suicidal behavior. Am J Med Genet B Neuropsychiatr Genet 150B: 799-807.
